# 

**Published:** 1900-06-23

**Authors:** 


					. ^rrixjv.cni.^ > A ilc 3iauua,u U1 t,igxiOT^ggRy.   ?u^ *oku, i?w.
CADBURY'S ^ H "fc* CADBURY'S
cocoa m m s^> cocoa
NO DRUGS OR ALKALIES USED.
Lasco?v Western Infirmar
No. 717.' Vol. xxvni. [?f2*?j?| Saturday.
June 23, 1900.
[SubscriptionTems,] pRICE TWOPENCE.

				

